# From hyperglycemia to intervertebral disc damage: exploring diabetic-induced disc degeneration

**DOI:** 10.3389/fimmu.2024.1355503

**Published:** 2024-02-20

**Authors:** Shuai Li, Jinpeng Du, Yunfei Huang, Shenglong Gao, Zhigang Zhao, Zhen Chang, Xuefang Zhang, BaoRong He

**Affiliations:** ^1^ Department of Spine Surgery, Honghui Hospital, Xi’an Jiaotong University, Youyidong Road, Xi’an, Shaanxi, China; ^2^ Medical College, Yan’an University, Yan’an, Shaanxi, China; ^3^ Shaanxi Key Laboratory of Spine Bionic Treatment, Xi’an, Shaanxi, China

**Keywords:** Intervertebral disc degeneration, high glucose, AGEs, cartilage endplates, nucleus pulposus, annulus fibrosus

## Abstract

The incidence of lumbar disc herniation has gradually increased in recent years, and most patients have symptoms of low back pain and nerve compression, which brings a heavy burden to patients and society alike. Although the causes of disc herniation are complex, intervertebral disc degeneration (IDD) is considered to be the most common factor. The intervertebral disc (IVD) is composed of the upper and lower cartilage endplates, nucleus pulposus, and annulus fibrosus. Aging, abnormal mechanical stress load, and metabolic disorders can exacerbate the progression of IDD. Among them, high glucose and high-fat diets (HFD) can lead to fat accumulation, abnormal glucose metabolism, and inflammation, which are considered important factors affecting the homeostasis of IDD. Diabetes and advanced glycation end products (AGEs) accumulation- can lead to various adverse effects on the IVD, including cell senescence, apoptosis, pyroptosis, proliferation, and Extracellular matrix (ECM) degradation. While current research provides a fundamental basis for the treatment of high glucose-induced IDD patients. further exploration into the mechanisms of abnormal glucose metabolism affecting IDD and in the development of targeted drugs will provide the foundation for the effective treatment of these patients. We aimed to systematically review studies regarding the effects of hyperglycemia on the progress of IDD.

## 1 Introduction

Low back pain (LBP) is a common public health concern worldwide (1, 2). Approximately 60-80% of patients experience chronic low back pain throughout their lifetime (3–5). Furthermore, LBP is the main cause of disability and productivity loss and seriously affects the quality of life of patients (6, 7). Although the cause of most cases of LBP is unclear, intervertebral disc degeneration (IDD) is considered to be the most common factor. The intervertebral disc (IVD) consists of the annulus fibrosus (AF), nucleus pulposus (NP) and cartilage endplate (CEP) (8–10). The NP is rich in proteoglycans and type II collagen and is highly hydrated. Therefore, physiological osmotic pressure can easily dissipate any mechanical force transmitted through the spine (11, 12). The AF is a layered structure mainly composed of type I collagen (13, 14), whereas the CEP is composed of transparent cartilage located between IVD soft tissue and the vertebral bone structures (15, 16). The CEP is crucial in the maintenance of mechanical integrity and nutrient exchange of the IVD. Furthermore, the IVD can increase the range of spinal movement, withstand pressure, cushion vibration and protect the spinal cord (17).

**Figure 1:**
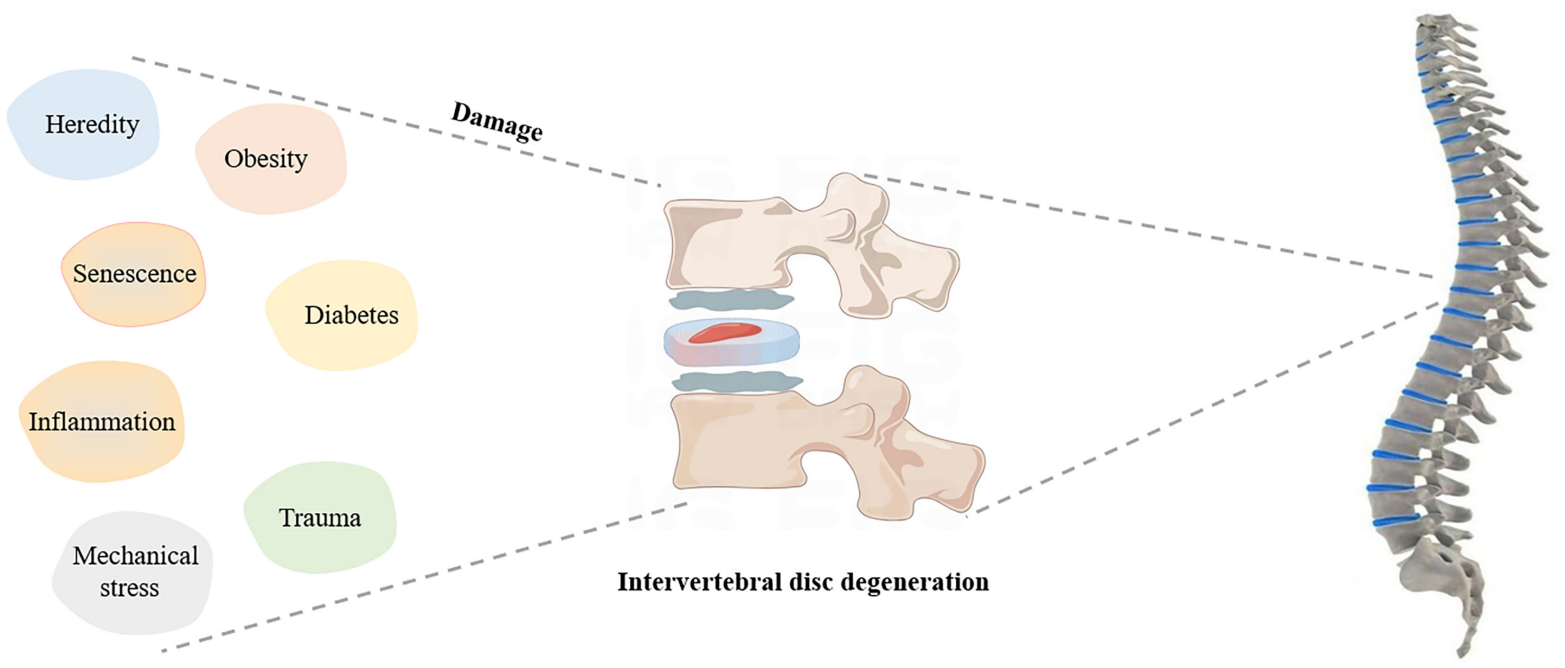
Possible factors affecting IDD.

Degeneration of the IVD occurs naturally with age, weakening its elasticity and toughness (18, 19). Imbalance between anabolism and catabolism in the IVD can lead to changes in the composition of the extracellular matrix (ECM), cell loss, excessive oxidative stress and inflammation (20, 21). Additionally, abnormal spinal mechanical changes can easily cause a series of symptoms of intervertebral disc herniation. Age, repeated mechanical stress, occupational factors, metabolic disorders (such as obesity and diabetes), trauma, heredity, and even smoking may lead to the development of IDD (22–25). The possible factors affecting IDD are described in the **Figure 1**.

## 2 Current treatment of IDD

### 2.1 Conservative treatment

The preferred treatment methods for lumbar and leg pain caused by IDD are lifestyle changes, encompassing weight management (reducing body weight as a means to alleviate spinal pressure), optimizing posture (acquiring and sustaining proper standing, sitting, and lifting postures), incorporating regular breaks (averting prolonged static positions and periodically altering postures), thermal therapy (alternating between cold and heat applications to mitigate pain and inflammation), refining sleep quality (utilizing appropriate mattresses and pillows, discovering sleep positions conducive to alleviating spinal pressure), spinal traction (alleviating pain and diminishing intervertebral disc pressure), acupuncture, and massage (inducing muscle relaxation and alleviating discomfort) (26, 27). Lifestyle changes have a significant effect. Physiotherapy also plays a vital role in relieving the symptoms of IDD. On the drug therapy aspect, opioids, steroids, and non-steroidal anti-inflammatory drugs play an important role in controlling pain and improving function and quality of life (28–30). While medications prove effective in managing pain and inflammation, prolonged use carries potential side effects, with opioids posing a risk of dependence. Emphasis must be placed on the crucial need to customize treatment plans based on individual patient needs and responses. Moreover, the sustained efficacy of these conservative measures varies within patient populations, underscoring the imperative for personalized therapeutic approaches that consider specific patient circumstances, comorbidities, and responses to initial treatments.

### 2.2 Surgery

Surgical treatment includes discectomy, fusion, and total disc replacement, and can be considered as the gold standard of IDD treatment (31, 32). The decision for surgical intervention in cases of IDD is a complex and multifaceted process that requires a comprehensive evaluation, combining imaging studies such as CT, MRI, and bone density scans with an assessment of the patients overall physical condition. Typically, surgery becomes a pivotal consideration when conservative treatments, including medication and physical therapy, prove inadequate in providing substantial relief from symptoms. The presence of neurological symptoms such as numbness, muscle weakness, or difficulty in walking signifies compression of nerve roots or the spinal cord, serving as a critical determinant for opting for surgical decompression procedures. Additionally, the patients general health, encompassing coexisting conditions like diabetes, osteoporosis, or cardiovascular diseases, along with the patients willingness to undergo surgery, are pivotal factors influencing the decision for surgical intervention. Surgery can effectively relieve the symptoms of nerve compression and prevent the subsequent deterioration of muscle strength (33). With the development of three-dimensional (3D) printing technology and synthetic biomaterials, using artificial intervertebral discs to replace degenerative IVD has gradually become feasible (34–36). In summary, the decision to undergo surgical intervention should be guided by the substantial benefits observed in terms of pain alleviation, functional enhancement, and overall improvement in the quality of life during the evaluation of the patient. Surgical recommendation is warranted when there is a discernible and significant positive impact in these critical aspects.

### 2.3 Biotherapy

IDD is a multi-factor process. Biomolecule therapy aims to target disease states characterized by decreased anabolism and increased catabolism (37–39). A variety of biotherapies have been evaluated in preclinical and clinical studies, including the use of growth factors and platelet-rich plasma (18, 40, 41). Biomolecule therapy focuses on repair due to the loss of proteoglycans and collagen, and its goal is to reduce the environment of pro-inflammatory catabolism and anti-anabolic metabolism in IDD.

### 2.4 Cellular and acellular therapy

Cell therapy is one of the best treatment strategies for patients with mid-term IDD, playing plays an active role by increasing the number of normal cells in the diseased IVD. Cell transplantation can secrete various cytokines, immune receptors, and anti-inflammatory molecules to regulate the microenvironment of host tissues (42, 43). Furthermore, extracellular vesicles (EVs), as acellular structures, can freely shuttle between cells and perform signaling and communication functions (44–46). EVs regulates apoptosis, senescence, proliferation and inflammation by delivering targeted non-coding RNA and various proteins to recipient cells (47, 48).

## 3 Abnormal glucose metabolism and IDD

**Figure 2:**
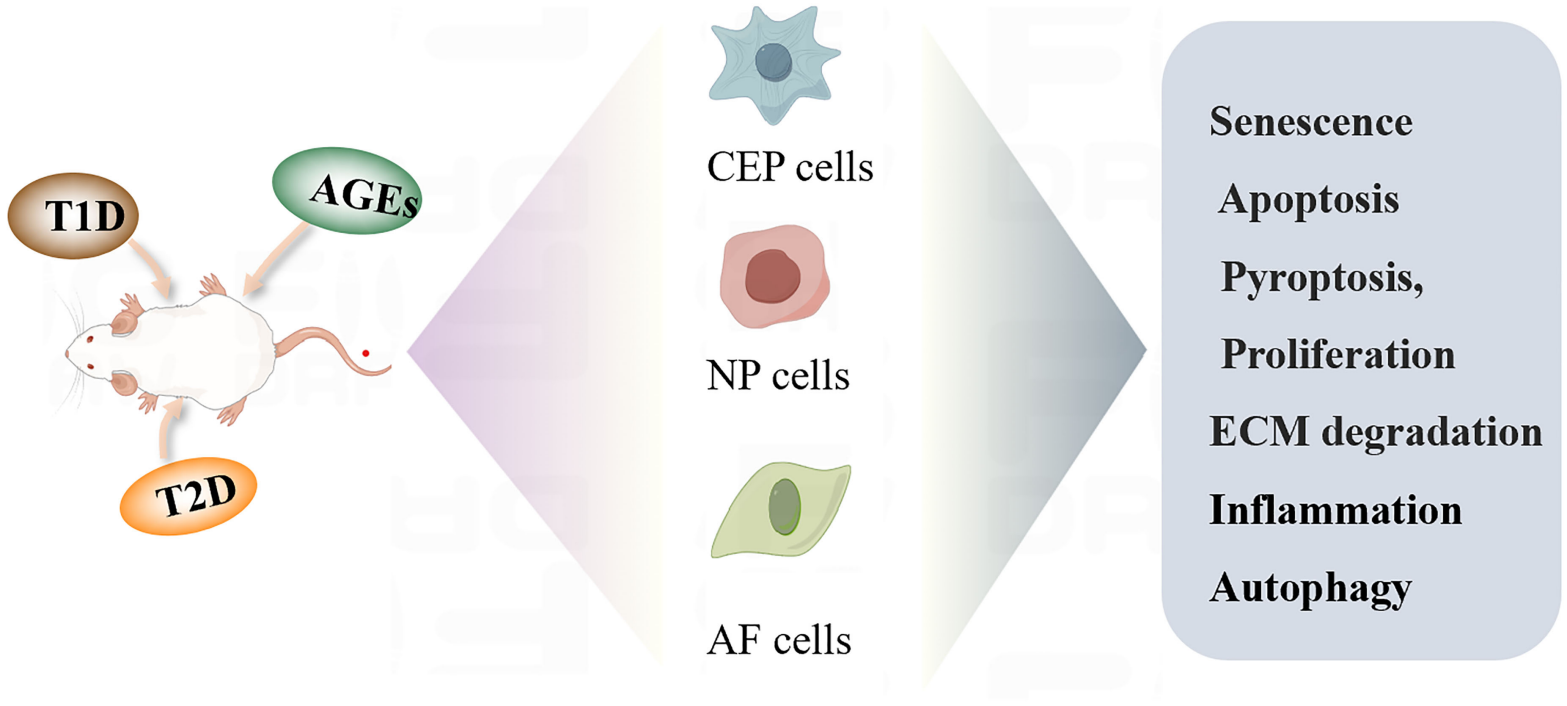
Diabetic IDD regulates the biological function of cells.

In addition to abnormal mechanical overload, abnormal fat accumulation, changes in glucose and lipid metabolism and inflammation are important factors affecting IVD homeostasis and pro-inflammatory pathophysiology (49–51). High concentrations of pro-inflammatory cytokines, adipokines, sugars, and lipids, aggravate systemic low-grade inflammation, which consequently impairs the metabolism of articular chondrocytes and IVD cells. Abnormal glucose metabolism is an important adverse factor in triggering and exacerbating IDD, and various biological processes and mechanisms of abnormal glucose metabolism affect cell senescence, apoptosis, inflammation, proliferation, and ECM degradation in IDD (**Figure 2**).

### 3.1 Type 1 diabetes induced IDD

Type 1 diabetes (Non-obese diabetes, T1D) is an autoimmune disease characterized by destruction of pancreatic β cells, affecting more than 1.2 million children and adolescents worldwide. For these patients, daily insulin injections are necessary to maintain healthy blood sugar levels. Viral infection (enterovirus and coxsackievirus), streptozotocin, antibiotics and epigenetic modification are risk factors for the formation of T1D (52, 53). Ina clinical study, 118patients with T1D were retrospectively analyzed and evaluated by Pfirrmann score system and MRI. The results showed that IDD was serious in patients with poor blood glucose control. T1D can cause early degeneration of the IVD (54). At the cellular level, NP tissues from Streptozotocin (STZ) -induced T1D rats with IDD rats were collected for transcriptome sequencing and bioinformatics analysis. The results showed that BMP7 could be used as a core gene, and NLRP3 inflammasome and pyroptosis related markers were significantly increased in NP cells. Further experimental detection showed that BMP7 inhibits the activation of NLRP3 inflammasome and cell pyroptosis processes, thereby slowing down the IDD of T1D rats (55). In addition, in T1D mice, higher levels of ADAMTS were shown in the intervertebral disc matrix, which mediated glycan fragmentation and IVD apoptosis (56). These studies show that T1D is an important factor in promoting the progress of IDD, and that effective blood glucose control is the key strategy to delay IDD.

### 3.2 Type 2 diabetes induced intervertebral disc degeneration

Type II diabetes (T2D) is a chronic metabolic disease characterized by long-term hyperglycemia and insulin resistance (IR), and the decrease of insulin absorption and glucose utilization leads to more compensatory insulin secretion, resulting in hyperinsulinemia (57, 58). Long-term hyperglycemia can lead to decreased organ function due to various health complications (59, 60). A clinical observation showed that the height of the IVD decreased significantly in T2D patients, which may lead to an increase in the risk of vertebral fracture in T2D patients (61). In addition, increased oxidative stress and AGE/RAGE-mediated interaction may be important factors leading to an increase in the incidence of T2D IVD disease (62). Contrastingly, a diabetic mouse model was established by feeding with a HFD, and it was found that HFD mice showed obvious IDD. A microarray analysis showed that the differential genes ADAMTS4 and ADAMTS5 were increased in IDD samples. Overexpression of ADAMTS4 and ADAMTS5 accelerated the degradation of the ECM and led to the occurrence of IDD. In mechanism, the complex formed by CBP-PGC-1 α-Runx2 binds to the initiation binding regions of ADAMTS4 and ADAMTS5 to promote gene expression (63).

Leptin receptor deficient knockout (db/db) mice are mature models for the study of T2D. Leptin receptor deficiency can lead to cortical and trabecular bone changes, decrease torsional destruction strength, and destruction of biomechanical behavior of IDD (64). Furthermore, knockdown of the leptin receptor can increase the level of MMP3 and induce apoptosis (65). Moreover, Vitamin K is also related to glucose metabolism and insulin sensitivity. In IDD induced by T2D, vitamin K2 diets may exert an anti-inflammatory function by regulating Socs3 and Hmox1 (66). In summary, the prevention and treatment of IDD induced by T2D cannot be ignored. The current research is still limited, and further exploration is needed to elucidate the mechanism of high glucose-induced IDD. Both Type 1 and Type 2 diabetes contribute to the exacerbation of IDD, albeit with slightly distinct mechanisms. Owing to an earlier onset and its autoimmune nature, T1D may precipitate an earlier onset of IDD with potentially accelerated progression. In contrast, IDD linked to T2D may manifest more covertly, exacerbated by the emergence of metabolic syndrome. For T1D, a concentrated effort on stringent blood glucose control and vigilant monitoring of early signs of IDD may prove beneficial. In the context of T2D, addressing the pervasive influence of metabolic syndrome and achieving optimal blood glucose control may be more efficacious in IDD management. In essence, although both diabetes types exert adverse effects on IDD, an in-depth comprehension of these mechanisms will facilitate the development of more targeted and efficacious treatment approaches for IDD within the diabetic milieu.

### 3.3 Advanced glycation end and IDD

The continuous increase of blood glucose levels in patients with diabetes can lead to non-enzymatic macromolecular glycosylation and eventually to the formation of advanced glycation end products (AGEs) (67, 68). AGEs accumulation is the source of complications of diabetes. AGEs are mainly accumulated in proteins, including aggregating glycans and collagen (69, 70), causing the fibers to soften, rendering the dehydrated matrix unable to withstand the typical mechanical forces of the spine, resulting in biomechanical changes (71). Studies have determined the direct correlation between AGEs intake and the incidence of IDD. The NP samples of clinical IDD patients were detected, and it was found that compared with non-diabetic IDD patients, the level of AGEs in diabetic patients was significantly higher than that in non-diabetic controls (72). Transcriptome sequencing analysis showed that the expression of several MMP genes increased in IVD mice fed with HFD. AGEs treatment promotes the rise of the AP1-p300/CBP-PPRC pathway, and AGEs via PAK1/USP24/PPRC1-p300/CBP-AP1 signaling drives MMP to induce ECM degradation, which leads to IDD. Inhibition of this pathway by injection of inhibitors *in vivo* can effectively prevent the degeneration of the IVD (73). After ACG activation, Gal3 and RAGE can act as receptors of ACG to affect collagen, in which Gal3 has a protective effect on AGE attack, limiting collagen damage and biomechanical changes. RAGE is an essential receptor for collagen injury induced by AGEs (74). MRI image analysis showed that oral treatment with the AGE inhibitor PM could reduce NP dehydration after needle injury. PM also promoted the increase in the level of oligosaccharides in rats (75). In addition, AGEs-BSA can induce mitochondrial damage in NP cells through activation of NLRP3 inflammatory bodies, resulting in a secondary inflammatory response (76). High AGEs content diets increase vertebral cortical thickening and endplate calcification, decrease IVD height and GAG content, and increase COL-X expression, which promotes the hypertrophic differentiation of NP cells. This accelerates aging of the spinal structures (77). In AF cells, AGEs affects cell viability and proliferation by regulating mitochondrial apoptosis and ROS production in a dose-dependent manner (78). The formation of AGEs requires sustained hyperglycemia, so there is no change in AGEs during a short duration of hyperglycemia (79).

## 4 High glucose induced cartilage endplate injury

The CEP is composed of chondrocytes and matrix, located at the upper and lower edges of the vertebral body, and proteoglycan and type II collagen (80). The main function of the CEP is to distribute the mechanical load along the spine to protect the vertebrae from compressive vertebral atrophy under pressure and to provide nutrition for the IVD (81). After high glucose induction, the mitochondrial membrane potential of CEP cells decrease and ROS production and apoptosis related proteins increased. High glucose may cause damage to the CEP cells through oxidative stress and CEP apoptosis induced by high glucose is time-dependent (82). Moreover, lncRNA MALAT1 play an important role in promoting apoptosis of rat CEP cells by activating the p38/MAPK signal pathway. This suggests that MALAT1 can be used as a therapeutic target for diabetes related IDD (83).

## 5 High glucose induced nucleus pulposus injury

The NP is located between the CEP and the AF, and is an elastic translucent gelatinous material composed of a crisscross fibrous reticular structure (84–86). The water content of the NP in infants is 80-90%. With an increase in age, the proteoglycan depolymerization in the NP increases, the water content decreases gradually and the collagen thickens and is gradually replaced by fibrocartilage (87, 88). Therefore, the incidence of IVD herniation in the elderly is significantly higher than that in young adults (89, 90).

The nutrition of the NP is mainly supplied by the vertebral body-cartilage endplate (84). During spinal movement, the NP rolls like a bearing to support the vertebral body and assists other parts of the spine to complete physiological activities (91). When the NP bears an external force, the force is uniformly transferred to the surrounding AF, which can balance the stress (86, 92). In a model of diabetes induced by streptozotocin injection, the protein levels of caspase-8, caspase-9, and caspase-3 apoptosis proteins in NP cells and the expression of aging marker p16lnk4A protein were significantly increased. In addition, the activation of the autophagy pathway in NP cells inhibits the expression of MMP-13 in diabetic IDD and triggers the protective mechanism of the NP cells (93, 94).

On the other hand, the addition of osteogenic protein-1 (OP-1) reverses NP matrix catabolism induced by high glucose (95). A high glucose concentration significantly decreases the proliferation, colony formation, migration, wound healing and dry maintenance of NP mesenchymal stem cells (NPMSCs) and accelerate the apoptosis and senescence of NPMSCs (96). In recent years, stem cell therapy has been widely used in cell-based regenerative medicine (97–99). Treatment of NPMSCs with hMSCs-CM inhibits the level of phosphorylated p38 MAPK (100). Autophagy has been shown to prevent premature aging under various conditions, and autophagy defects may lead to aging (101, 102). Through screening diabetes data -sets and tissue validation, it was found that HuR expression in diabetic NP tissue and high glucose treated NP cells was reduced. Knockdown of HuR promotes senescence of the NP cells. Further exploration of the mechanism shows that HuR can bind to the key autophagy gene Atg7 and regulate the stability of Atg7. *In vivo* injection of Atg7 overexpression improves IDD progression by promoting autophagy (103). It is of great significance to explore new methods to prevent IDD and cell senescence from the point of view of the NP and NPMSCs.

## 6 High glucose induced annulus fibrosus injury

As the external structure of the IVD, the AF is used to protect the NP from being released from IVD during axial compression, tension and bending when the spine is under high load. The AF can maintain physiological intervertebral disc pressure. When AF cell degeneration also occurs, various pathological events may occur, including decreased IVD cells, upregulation of matrix degrading enzymes, and inflammation. Studies have shown that AF cells of young rats treated with high glucose for 1 and 3 days showed increased mitochondrial damage and increased cell senescence markers, excessive production of ROS and decreased average telomerase activity (104). Stress-induced accelerated premature senescence of young AF cells may be an emerging risk factor for premature disc degeneration in young DM patients. In addition, high glucose culture significantly increase the caspase-3 and caspase-9 activity of AF cells and promotes the activation of the apoptosis pathway. In mechanism, inhibition of JNK pathway and p38 MAPK signal pathways may attenuate the effect of high glucose on apoptosis of the AF cells (105). Furthermore, endoplasmic reticulum stress can lead to the disorder of steady-state behavior of many cells and induce IVD apoptosis to degenerate. After high glucose induction, the protein expression of ER stress markers CHOP, ATF-6, and ATF-6 significantly increased and induced apoptosis in the AF cells (106).

## 7 Drug therapy of Diabetic IDD

Surgery can directly and effectively solve the pain and neurological symptoms of patients, but it is not helpful for the prevention and progress of IDD. Therefore, effective drugs should be developed to intervene in the early stage of IDD or to prevent IDD in high-risk groups, to avoid trauma and risk of surgical treatment for patients. Research has found that adding resveratrol to high glucose induced NP cells can reduce apoptosis, ROS production, downregulate the expression of aging markers (p16 and p53), and increase telomerase activity and the expression of anti-apoptotic molecules (Bcl-2) in NP cells. On the contrary, inhibition of the PI3K/Akt pathway can counteract the beneficial effect of resveratrol in high glucose groups (107).

Glucagon-like peptide-1 (GLP-1) is a key intestinal insulin-stimulating hormone that regulates glucose and energy homeostasis (108, 109). Liraglutide (LIR), as a long-acting GLP-1 analog, is highly homologous to endogenous GLP-1 (110). LIR is considered to be a powerful therapeutic choice for T2D by binding to GLP-1R to regulate insulin and cell proliferation, differentiation and apoptosis (111, 112). In diabetic IDD, the addition of high concentration of LIR inhibited the apoptosis of NPs induced by high glucose, increasing cell activity and proliferation. The PI3K/Akt/caspase-3 pathway may be one of the mechanisms by which LIR plays a protective role in IDD (113, 114).

Marein is a flavonoid extracted from C. tinctoria, which can improve insulin resistance induced by high glucose levels. In IDD, Marein protects HNPC from HG-induced damage and ECM degradation by inhibiting the ROS/NF-κB pathway. Therefore, Marein is a promising therapeutic agent to delay IDD in patients with diabetes (115).

Cycloastragenol is the only telomerase activator that has anti-aging effects by increasing telomerase levels to delay telomere shortening (116, 117). Astragaloside IV (AG-IV) has a variety of drug properties, such as anti-inflammatory, anti-IR and neuroprotective effects (118–120). High glucose concentration is known to have adverse effects on telomerase reverse transcriptase (TERT) expression and telomere length in NPs. After treatment with CAG and AG-IV, the cell morphology and vitality significantly improve, the TERT expression of NPC and telomere length increased, and apoptosis and senescence an inhibited (121). At present, the exploration of novel pharmacotherapeutic strategies for treating IDD, particularly in the presence of concurrent diabetes, represents a highly promising avenue. Compounds such as resveratrol, liraglutide, and Marein exhibit diverse mechanisms of action, underscoring the potential of targeted drug interventions in mitigating IDD. It is crucial to comprehend how these drugs interact with conventional diabetes medications and their impact on blood glucose control. Longitudinal studies are imperative to assess the prolonged safety and efficacy of these drugs in IDD patients, especially considering the chronic nature of IDD and diabetes. The selection of pharmacological interventions should be personalized based on the individual patients circumstances, the stage of IDD, and the status of diabetes.

## 8 Summary and prospects

Studies have shown that both T1D and T2D can cause different degrees of IDD, and that high glucose and HFD are risk factors for diabetic IVD. High glucose can promote the aging of endplate cells, NP cells, and AF cells, increase cell apoptosis and inflammatory response, and hinder the formation of autophagy pathways and ECM degradation. The relevant mechanisms are described in **Table 1**.

**Table 1:** Mechanisms involved in the progress of Diabetic IDD.

Names	Signaling pathway	Function	Ref
AGEs	PAK1/USP24/PPRC1-p300/CBP-AP1-MMP axis	Promote ECM degradation	(1)
STZ	BMP7-NLRP3 axis	Promote pyroptosis	(2)
High glucose	CBP-PGC-1α-Runx2 complex-ADAMTS4/5 axis	Promote ECM degradation	(3)
High glucose	ChREBP/p300- Puma /BAX axis	Promote cell apoptosis	(4)
Resveratrol	ROS-PI3K/Akt axis	Inhibit cell apoptosis and senescence	(5)
LIR- GLP-1R	PI3K/Akt/mTOR/caspase-3 PI3K/Akt/GSK3β/caspase-3	Inhibit cell apoptosis	(6)
High glucose	HuR-Atg7 axis	Suppress cell senescence	(7)
Marein	ROS-NF-κb axis	Inhibit ECM degradation	(8)
High glucose	JNK - p38 MAPK axis	Promote cell apoptosis	(9)
AGEs	BRD4- MAPK/NF-κB -MMP-13 axis	Inhibit ECM degradation	(10)
AGEs-	NF-κB -NLRP3 axis	Promote inflammation	(11)
LncRNA MALAT1	p38 MAPK	Promote cell apoptosis	(12)

It is worth noting that some drugs have shown beneficial effects on alleviating high glucose induced IDD, but current drug therapy is still limited. We believe that health education for the population is the preferred strategy to preventing high glucose induced IDD in the future. Patients who adhere to a reasonable diet and regular living habits can effectively alleviate the progress of IDD. In addition, it is necessary to further reveal the mechanism of high glucose-induced IDD and develop specific targeting drugs. Drugs enter the body through blood circulation and are mostly metabolized by the liver and kidney. The effective dose to achieve the function of the IVD is usually greatly reduced. Therefore, the application of nanoparticles to encapsulate drug components and enable drugs to target the therapeutic area should be considered. For example, exosomes, EVs, and artificial vesicles can all serve as good carriers for drugs. In summary, current research provides a fundamental basis for the treatment of high glucose induced IDD patients. With the further exploration of more mechanisms of abnormal glucose metabolism affecting IDD and in the development of targeted drugs, it will bring dawn to the rehabilitation of patients.

## Author contributions

SL: Writing – original draft. JD: Data curation, Writing – original draft. YH: Software, Writing – original draft. SG: Software, Writing – original draft. ZZ: Data curation, Writing – original draft. ZC: Investigation, Software, Writing – original draft. XZ: Formal Analysis, Funding acquisition, Software, Writing – original draft. BH: Supervision, Writing – review & editing.
